# Protein Tyrosine Phosphatase Gamma (PTPγ) is a Novel Leukocyte Marker Highly Expressed by CD34^+^ Precursors

**Published:** 2007-05-31

**Authors:** Andrea Mafficini, Marzia Vezzalini, Loris Zamai, Laura Galeotti, Gabriella Bergamini, Marco Della Peruta, Paola Melotti, Claudio Sorio

**Affiliations:** 1 Department of Pathology, University of Verona, Italy; 2 Institute of Histology and Laboratory Analysis, University of Urbino “Carlo Bo”, Italy; 3 Flow Cytometry and Cytomorphology Center, University of Urbino “Carlo Bo”, Italy; 4 INFN-Gran Sasso National Laboratory, SS17bis km 18+910, 67010 Assergi, L’Aquila, Italy; 5 Cystic Fibrosis Center, Azienda Ospedaliera of Verona, Verona, Italy

**Keywords:** Egg yolk immunoglobulin (IgY), human protein tyrosine phosphatase gamma (PTPγ), fluorescence activated cell sorting (FACS), haematopoietic progenitors

## Abstract

Protein Tyrosine Phosphatase gamma (PTPγ) is a receptor-like transmembrane protein belonging to the family of classical protein tyrosine phosphatases. PTPγ is known to regulate haematopoietic differentiation in a murine embryonic stem cells model. We have recently demonstrated that PTPγ mRNA is expressed in monocytes, tissue-localized myeloid dendritic cells and in both myeloid and plasmacytoid dendritic cells in peripheral blood. We now developed a PTPγ specific antibody that recognizes the protein by flow cytometry. PTPγ expression was detected in monocytes and both myeloid and plasmacytoid dendritic cells, while PMN showed a low but consistent staining in contrast with previous mRNA data. B cells were found to express the phosphatase while T cells were negative. In keeping with RNA data, PTPγ was detected in monocyte-derived dendritic cells and its expression rose upon LPS stimulation. Finally, we discovered that CD34^+^ haematopoietic precursors express high PTPγ level that drops during *in vitro* expansion induced by IL-3 and SCF growth factors. We therefore propose PTPγ as a new functionally regulated leukocyte marker whose role in normal and pathological context deserve further investigation.

## Introduction

Protein phosphatases, together with kinases, control critical aspect of cellular signaling as they can modify the phosphorylation level of the cell, leading to pleiotropic effects on proliferation, differentiation and survival (Andersen et al. 2001). Phosphatases can be divided in several families according to their substrate specificity. Protein tyrosine phosphatases (PTPs) are further divided in two groups. Classical PTPs have phosphotyrosine as exclusive substrate, while dual specificity phosphatases have a more open active site cleft, allowing them broader substrate specificity (Tonks, 2003). PTPgamma (PTPγ) is a receptor-like transmembrane protein belonging to the family of classical PTPs; these enzymes can exist in transmembrane (receptor-type PTPs, RPTPs) or non-transmembrane form (described in http://ptp.cshl.edu/). RPTPs can be classified in nine subtypes, according to the combination of structural motifs featuring in the N-terminal moiety; PTPγ belongs to the subtype V, characterized by the presence of a carbonic anhydrase-like and a fibronectin type III domain at the N-terminus (Barnea et al. 1993). PTPγ has been proposed as a tumor suppressor gene whose expression is lost in various neoplastic diseases including renal cell carcinoma, lung, ovarian, breast and colorectal cancers (LaForgia et al. 1993, Lubinski et al. 1994, Tsukamoto et al. 1992, van Niekerk and Poels, 1999, Liu et al. 2004, Wang et al. 2004, Vezzalini et al. 2007). Involvement in haemopoietic neoplasms has also been reported (van Doorn et al. 2005, Vezzalini et al. 2007). These data suggest a role for PTPγ in the molecular pathways that regulate proliferation and differentiation. Indeed, it has been shown that PTPγ is able to regulate haematopoietic differentiation in a murine embryonic stem cells model (Sorio et al. 1997). We have recently demonstrated that PTPγ is expressed in peripheral blood myeloid and plasmacytoid dendritic cells as well as in monocytes where it is differentially regulated during *in vitro* differentiation to dendritic cells or macrophages (Lissandrini et al. 2006). These latest findings suggested the possibility that PTPγ represents a novel marker for myeloid cells in the haemopoietic system. Suitable antibodies that can be used in conjunction with lineage-restricted markers are not available. We recently developed a new chicken antibody targeted against the extra cellular domain of PTPγ suitable for flow cytometric analysis. Using this newly developed tool, we investigated the expression of this phosphatase in cell lines, peripheral blood samples and purified haemopoietic precursors.

## Materials and Methods

### Cells, purification and culture

The cell lines K562 (Andersson et al. 1979), HL-60 (Gallagher et al. 1979), THP-1 (Tsuchiya et al. 1980) and U937 (Sundstrom and Nilsson, 1976) were purchased from ATCC, ML-3 (Ohyashiki et al. 1986) was a kind gift from prof. M.A. Cassatella. Circulating human monocytes (>70% pure as assessed by expression of CD14), polymorphonuclear cells (>95% pure as assessed by CD15 expression) and lymphocytes (>95% pure as assessed by morphology and the lack of CD14) were purified by Percoll (Pharmacia Uppsala, Sweden) gradient centrifugation from leukocyte-rich buffy coats obtained from human blood of healthy donors, as described elsewhere (Muzio et al. 1994). Immature monocyte-derived human dendritic cells (moDC) were obtained *in vitro* as previously described (Sozzani et al. 1997). Briefly, purified monocytes were cultured in RPMI 1640 (2 × 10^6^ cells/well) containing 10% heat-inactivated FCS, 2mM glutamine and supplemented with 50 ng/ml recombinant human GM-CSF and 20 ng/ml recombinant human IL-4 (Peprotech, Rocky Hill, NJ) for 5–6 days. To induce maturation, immature moDC were treated for 24 h with 100 ng/ml LPS (Escherichia coli serotype 026: B6; Sigma, St. Louis MO) cells were subjected to flow cytometry analysis to assess the activation/maturation status as previously described (Lissandrini et al. 2006). For CD34^+^ cell selection, mononuclear cells were isolated from healthy donors buffy coats by Ficoll/Histopaque-1077 (Sigma-Aldrich, Milan, Italy) gradient centrifugation, rinsed and adherence-depleted for one hour as described in (Zamai et al. 2000) with modifications. After removal of adherent cells, CD34^+^ cells isolation was accomplished by immunomagnetical positive selection using magnetic beads coated with anti-CD34 mAb (CD34 isolation kit plus Vario-MACS system, Miltenyi Biotech, Bologna, Italy), according to the manufacturer’s instructions; cells were subjected to flow cytometry to assess purity and PTPγ expression. Purified CD34^+^ cells (50,000/ml) were cultured for 3 days in 1 ml X-Vivo 20 (BioWittaker, Walkersville, MA, U.S.A) medium supplemented with 10% human AB serum in the presence of stem cell factor (SCF, 50 ng/ml) and IL-3 (10 ng/ml). All cytokines were purchased from Peprotech EC Ltd. (London, U.K.).

### Antibody production

A chicken polyclonal antibody was raised against the sequence CZ NED EKE KTF TKD SDK DLK (residues #390–407 of extra cellular PTPγ sequence); produced IgY were affinity purified against the same sequence by Aves Labs (Tigard, OR, U.S.A). Pre-immune chicken IgY were collected and purified from the same hen before the immunization process. Briefly, the antigen was injected into the pectoral muscle of one American-laying hen. Three booster injections containing 50 μg of antigen mixed with incomplete Freund’s adjuvant were given at 2, 4 and 6 weeks. The eggs were collected daily and stored at 4 °C until the antibodies were extracted. The crude anti-PTPγ chicken IgY (chPTPγ) was further purified using affinity column chromatography against the 20-amino acid peptide. The non-specific proteins were washed from the column with washing buffer (0.1 M Tris–HCl, 0.5 M NaCl, pH 8.0), until the absorbance at 280 nm decreased to zero and the antibody was then eluted with a desorbing agent (0.1 M glycine, pH 3.0)

### PTPγ extra cellular domain-enriched supernatants production

293F cells (Invitrogen, Milan, Italy) were transfected with a cDNA encoding for the extra cellular domain of PTPγ (pPTPx), containing the epitope of interest, cloned in pRC/CMV vector (Invitrogen, Milan, Italy). Transfection was achieved using Lipofectamine2000^™^ (Invitrogen, Milan, Italy), diluted in OptiMEM^™^ (Invitrogen, Milan, Italy), according to the manufacturer’s optimized protocol for 293F cells. Positive cells were selected by plating transfected samples at about 10^5^ cells/mL in RPMI 1640, 10% FBS, 1% Ultraglutamine, 0.9 mg/mL G418 (Invitrogen, Milan, Italy) and incubating them in a humidified atmosphere with 5% CO_2_ at 37 °C. Exhaust medium was harvested every 48 hours and centrifuged 5 min at 350 × g to eliminate residual cells. After decanting, the supernatant was further centrifuged for 10 min at 3000 × g to avoid cellular debris. The clarified pools were stored at −20 °C until needed.

### Western blot analysis

Equal amount of serum-free spent medium were mixed to 0.3 volumes of sample buffer (160 mM Tris, 20% Glycerol, 5% β-mercaptoethanol, 4% SDS, 0.01% bromophenol blue) and loaded on a 10% polyacrylamide, 0.1% SDS gel. Gels were run for 1 hour at 200V, 14 mA in a Mini Protean 3 Apparatus (Biorad, Milan, Italy) with 0.3%Tris, 1.45% Glycine, 0.1% SDS running buffer, pH 8.9. After SDS-PAGE, gels were incubated for 20 min in transfer buffer (SDS running buffer, 20% methanol) and then electro-blotted on Hybond nitrocellulose (GE Healthcare, Milan, Italy) for 1 hour at 100V, 120 mA in a Mini Trans-Blot Electrophoretic Transfer Cell (Biorad, Milan, Italy); the homogeneous transfer of the samples on membranes was checked by Ponceau staining. For use with chPTPγ, membranes were saturated by incubation for 1 hour in 10% BlockHen II™ (AvesLabs, Tigard Oregon, U.S.A). After three washes in PBS, 0.05% tween^®^ 20 the membranes were incubated overnight with chPTPγ (1μg/ml) diluted in PBS, 0.1% tween^®^ 20. Membranes were then washed three times with PBS, 0.05% tween^®^ 20 and incubated for 1 hour with goat anti-chicken HRP (AvesLabs, Tigard Oregon, U.S.A; dilution 1:15,000). After three further washes, membranes were assayed with ECL (GE Healthcare, Milan, Italy).

### RNA extraction and northern blot

10 μg of total RNA was extracted from cell lines using the TRIzol^®^ reagent (Invitrogen, Milan, Italy) according to the manufacturer instructions and electrophoresed on a 1% formaldehyde-agarose gel, blotted onto a Hybond N+ membrane and hybridized to ^32^P-labeled cDNA probes prepared by random priming kit (Ready to-go DNA labeling Beads, Amersham Pharmacia Biotech, Uppsala, Sweden) using α-^32^P dCTP (ICN, Costa Mesa, CA). The hybridization was performed as described (Sambrook et al. 1989). PTPγ, PTPζ, CD148 and actin cDNA were used as probes. PTPγ cDNA was amplified by PCR from PTPγ pCR^®^3.1 plasmid (Invitrogen) using the following primers: forward 5′ CGT CAC CAG TCT CCT 3′, reverse 5′ GAA GAG GCA GGA GAG 3′. PTPζ cDNA probe was obtained by reverse transcription of total RNA from human astrocytoma tissue and amplified using the following primers: forward 5′ CTA GCT GAG GGG TTG GAA TC 3′, reverse 5′ GTG CCT GTT CTT CCA ACT CC 3′. CD148 cDNA probe was obtained by reverse transcription of total RNA from human peripheral blood granulocytes and amplified using the following primers: forward 5′ TGC CAC ACA AGG ACC 3′, reverse 5′ TGA TTT GCT CCC CAC 3′. The identity of both probes was confirmed by sequencing.

### Flow cytometry of purified cells and whole blood

Purified cells and whole blood samples were stained with monoclonal anti-CD34 PE (clone AC136, Miltenyi Biotec, Bologna, Italy), anti-CD14 PE, anti-CD15 FITC, anti-CD3 PE, anti-CD19 PE, anti-CD1c biotin, anti-CD303 biotin, anti-CD1a FITC, anti-CD80 PE, anti-CD86 PE, anti-CD83 FITC (BioLegend, San Diego, CA) and the anti-PTPγ chicken IgY established in our lab. Biotinylated antibodies were detected with streptavidin PE/Cy5 (BioLegend, San Diego, CA). As an isotypic control, pre-immune IgY and matched IgG1-PE or IgG2a-PE (BioLegend, San Diego, CA) were used. Briefly, purified cells were first blocked with 10% v/v human serum (Invitrogen, Milan, Italy) for 15 minutes at RT, then incubated with chPTPγ (2 μg/10^6^ cells) for 1 hour at RT. After one wash with staining buffer (5% FBS in PBS, 0.1% NaN_3_), cells were stained with goat anti-chicken Alexa488 (Invitrogen, Milan, Italy) for 30 min at RT and/or with monoclonal antibodies for 15 min at RT. Where biotinylated antibodies were included, a further wash was necessary, followed by an incubation with streptavidin PE/Cy5 for 15 min at RT. Peripheral blood samples were first incubated with 2 μg chPTPγ or pre-immune IgY for 1 hour at RT, then subjected to erythrocytes lysis by addition of 20 volumes of erythrocytes lysis solution (0.886% NH_4_Cl, 0.1% KHCO_3_, 0.006% EDTA) and incubation for 10 min at RT with gentle agitation. After addition of 0.5 volumes of staining buffer and pelleting of cells, a further wash with staining buffer was performed. Cells were then stained with goat anti-chicken Alexa488 for 30 min at RT and/or with monoclonal antibodies as above described. Flow cytometry was performed on a Becton Dickinson FACScan^®^ flow cytometer. Analysis of flow cytometry data was performed with FCS Express V3 software (De Novo Software) and geometric mean fluorescence intensity (MFI) ratio between chPTPγ and isotype control antibodies has been used to evaluate PTPγ density of expression.

## Results

### PTPγ expression in selected cell lines and antibody validation

**Figure 1:**
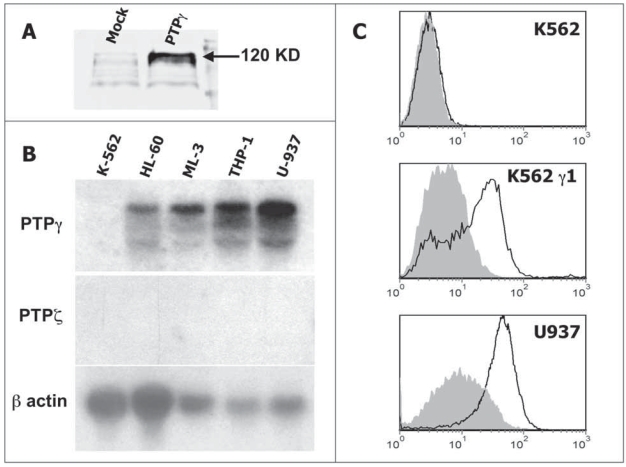
**A.)** Western blot analysis of conditioned media with chPTPγ antibody: Nctr = medium from 293 cells transfected with empty vector, PTPγ = medium from 293 cells transfected with PTPγ extra cellular domain cDNA, producing and secreting a soluble 120 KD protein. **B)** Northern blot analysis of haemopoietic cell lines for PTPγ; PTPζ is the other member of subtype V receptor type tyrosine phosphatase and is used as a negative control; β-actin is showed for total RNA estimation. As a positive control for PTPζ hybridization we utilized human brain RNA on the same blot (data not shown). **C)** FACS analysis of K562, K562 transfected with PTPγ full-length cDNA (K562 γ1) and U937 cell lines for PTPγ using chPTPγ antibody. Isotype control staining is showed in gray.

The capability to recognize PTPγ by the chicken antibody was evaluated by western blot on the recombinant extra cellular domain. We loaded conditioned media from 293F cells transfected with either an empty vector or with a cDNA for the expression of PTPγ extra cellular domain truncated at the level of the putative transmembrane region. The antibody specifically recognize the predicted 120 KD band in the gel lane loaded with the supernatant containing the recombinant extra cellular domain, being the supernatant derived from mock transfected cells negative. A similar, slight background was detectable in both the lanes (Fig. 1A). To validate the use of the antibody for flow cytometry we first analyzed PTPγ mRNA expression on a panel of cell lines by northern blot (Fig. 1B) and selected two cellular models: K562 completely lack PTPγ expression and was then transfected with both mock and PTPγ cDNA containing plasmids (K562 γ1). U937 was used as a positive control for native protein. As expected, chPTPγ specifically binds to U937 and K562 γ1 while K562 were negative (Fig. 1C).

### PTPγ is expressed in peripheral blood leukocytes

**Figure 2:**
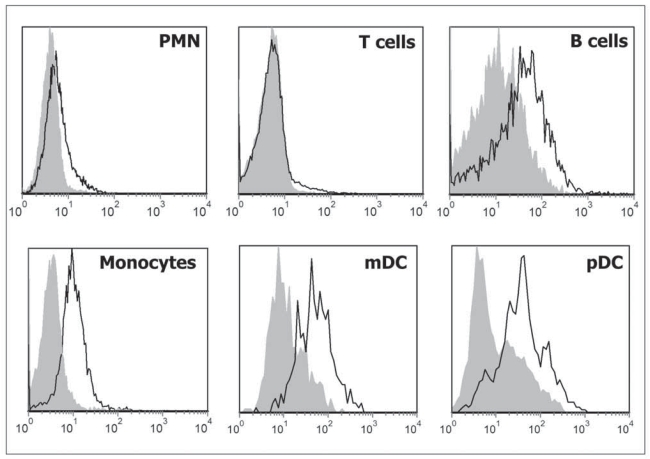
FACS analysis of peripheral blood PMN (CD15^+^; CD14^−^), T lymphocytes (CD3^+^), B lymphocytes (CD19^+^), monocytes (CD14^+^), myeloid (CD1c^+^/CD19^−^) and plasmacytoid (CD303^+^) DC using chPTPγ antibody. Isotype control is shown in gray, one representative experiment of eight from individual donors is shown.

We then checked PTPγ expression in peripheral blood leukocytes. Our previous work showed that purified monocytes express PTPγ mRNA, lymphocytes show a very low expression that we interpreted as residual (5%) monocytes contamination of the preparation, while polymorphonucleated cells (PMN) were negative (Lissandrini et al. 2006). Flow cytometry (Fig. 2) confirmed RNA data for monocytes. However individual staining of both B and T lymphocytes, obtained by CD19 and CD3 double staining, showed that B cells express detectable amount of PTPγ. Surprisingly, PMN showed a slight positive staining for PTPγ in contrast with RNA data. This signal was consistent and led us to hypothesize a residual expression of PTPγ protein on the surface of mature PMN in absence of active synthesis or its expression in a small subpopulation of granulocytes.

Staining of peripheral blood myeloid (CD1c^+^/CD19^−^) and plasmacytoid (CD303^+^) cells confirmed the results previously obtained by RNA analysis (Lissandrini et al. 2006), with plasmacytoid DC showing a slightly stronger staining than myeloid DC.

### PTPγ is expressed in monocyte-derived dendritic cells (moDC) and further induced by maturation

**Figure 3:**
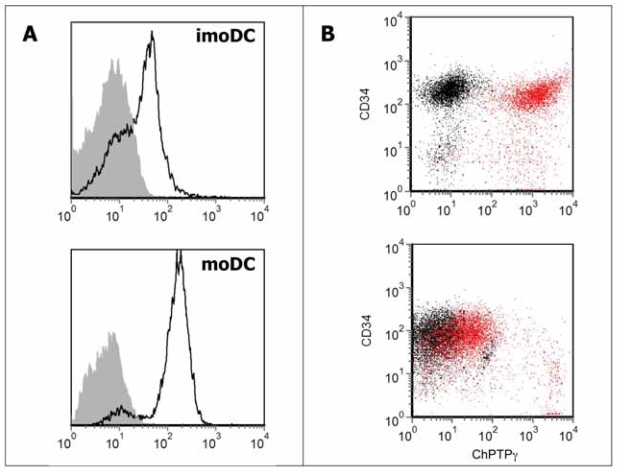
**A)** FACS analysis of immature (upper) or LPS-matured (lower) monocyte-derived DC using chPTPγ antibody; isotype control is shown in gray. **B)** double staining of purified peripheral blood precursors (upper) at the time of separation and after three days of culture in the presence of SCF and IL-3 (lower). In red: cells treated with anti-CD34 and chPTPγ; in black: cells treated with anti-CD34 and chicken isotype IgY. One representative experiment of four is shown. Each experiment involved purification, differentiation (for DC) and staining of cells from individual donors.

As moDC expressed high PTPγ levels we wished to confirm the expression and up-modulation of the protein upon induction of DC maturation by LPS. Cells were obtained from monocytes cultured in presence of IL-4 and GM-CSF and analyzed at day 6 of culture, after 24 h of incubation with or without LPS. In this case, flow cytometry with chPTPγ fully confirmed previous data, showing that moDC express the phosphatase during differentiation (geometric MFI ratio = 4.1) and that the expression is further enhanced upon LPS-induced maturation (geometric MFI ratio = 22.7) (Fig 3A).

### PTPγ is strongly expressed in purified CD34^+^ peripheral blood haematopoietic progenitors and down-modulated during *in vitro* expansion

We demonstrated that PTPγ expression cause a block in haemopoietic differentiation with an accumulation of cKIT/CD34^+^ precursors in murine ES cells (Sorio et al. 1997) and, more recently, described high PTPγ expression in endothelial cells (Vezzalini et al. 2007). We therefore wished to examine the presence of PTPγ on CD34^+^ cells, considered precursors for both haemopoietic and endothelial cells (Suda et al. 2000). We purified CD34^+^ cells from leukocyte-rich buffy coats and analyzed the expression of this phosphatase. Freshly isolated CD34^+^ cells showed the highest level of expression detected so far (geometric MFI ratio = 96.7 ± 9.75 SD, n = 4). After a three days treatment with SCF and IL-3 haematopoietic growth factors, known to induce proliferation and differentiation along erythro-myeloid lineage (Zamai et al. 2000), the levels of PTPγ expression decreases (geometric MFI ratio = 4.69 ± 1.50 SD, n = 4) (Fig. 3B).

## Discussion

The data here presented are aimed to complement those already exposed in our previous studies (Sorio et al. 1997, Lissandrini et al. 2006) regarding the expression of PTPγ in the haematopoietic system. The chPTPγ antibody recently developed by our group allowed us to move from mRNA expression data to surface labeling of cells and flow cytometry analysis. With few exceptions, the data confirmed at the protein level what had been seen at the RNA level: PTPγ is preferentially expressed by monocytes and dendritic cells. With the sole exception of PMNs the mRNA levels appear to correlate rather well with protein levels, indicating a translational control of the expression levels of this phosphatase. Moreover, its expression levels are substantially modulated when cells are induced to proliferate or differentiate. We also demonstrate that PTPγ is expressed by B cells that represent a class of lymphocytes with the capability to act as antigen-presenting cells together with classic myeloid APCs. CD34^+^ cells, precursors for both haemopoietic and endothelial cells (Suda et al. 2000), expressed PTPγ indicating that undifferentiated progenitors are characterized by expression of this phosphatase. There might be the possibility that PTPγ is required for the maintenance of the undifferentiated phenotype in haemopoietic precursors. This is suggested by previous data in the murine haemopoietic system where its overexpression inhibited erythro-myeloid differentiation with the accumulation of cKIT/CD34^+^ precursors (Sorio et al. 1997) and by the down modulation following *in vitro* culture with growth factors here observed. This latter finding, in agreement with its proposed tumor suppressor function, would suggest an inverse correlation between haematopoietic proliferation/differentiation and PTPγ expression on haematopoietic progenitor cells. Of course it must be considered that the high expression observed on freshly purified CD34^+^ cells might also be due to the purification procedure. Of note is the observation that all the anaplastic large cell lymphomas and Reed-Sternberg cells in Hodgkin’s disease stained positive for PTPγ expression while only 14% of B cell neoplasms were found positive (Vezzalini et al. 2007). This last result is of note given the expression detected in peripheral blood B cells and suggests different functions associated to specific lineages.

We recently found high PTPγ expression in endothelial cells (Vezzalini et al. 2007). The role of PTPγ in vascular biology is completely unknown even if endothelial cells are known to express many transmembrane PTPs involved in the regulation of intercellular contacts of vascular wall cells (Kappert et al. 2005). Our findings suggest a distinct role of PTPγ expression in the haemopoietic system, where its expression is reduced following the initial steps of maturation along the erythro-myeloid series, and endothelial cells where it is found strongly expressed in mature cells.

Altogether our data validate the use of a novel antibody for the detection of PTPγ. The results also indicate that PTPγ has the features of a novel leukocyte marker whose expression is modulated during differentiation and maturation of specific leukocyte subsets. Although more work is needed in order to elucidate the biological function of this phosphatase, the evaluation of its expression might represent a useful tool for the characterization of haematopoietic, endothelial and stem cells subsets.

## Abbreviations

IgY: Chicken egg yolk immunoglobulins
chPTPγ: Anti-PTPγ IgY antibody
PTPγ: human protein tyrosine phosphatase gamma
